# Crisis Reliability Indicators Supporting Emergency Services (CRISES): A Framework for Developing Performance Measures for Behavioral Health Crisis and Psychiatric Emergency Programs

**DOI:** 10.1007/s10597-015-9954-5

**Published:** 2015-09-29

**Authors:** Margaret E. Balfour, Kathleen Tanner, Paul J. Jurica, Richard Rhoads, Chris A. Carson

**Affiliations:** ConnectionsAZ, Phoenix, AZ USA; Department of Psychiatry, University of Arizona, Tucson, AZ USA; Connections SouthernAZ, Tucson, AZ USA; Crisis Response Center, 2802 E. District St., Tucson, AZ 85714 USA

**Keywords:** Mental health services/standards, Outcome and process assessment, Quality improvement, Emergency psychiatry, Crisis services, Behavioral health

## Abstract

Crisis and emergency psychiatric services are an integral part of the healthcare system, yet there are no standardized measures for programs providing these services. We developed the Crisis Reliability Indicators Supporting Emergency Services (CRISES) framework to create measures that inform internal performance improvement initiatives and allow comparison across programs. The framework consists of two components—the CRISES domains (timely, safe, accessible, least-restrictive, effective, consumer/family centered, and partnership) and the measures supporting each domain. The CRISES framework provides a foundation for development of standardized measures for the crisis field. This will become increasingly important as pay-for-performance initiatives expand with healthcare reform.

## Introduction

Crisis and emergency psychiatric services are an integral part of the behavioral health system of care, yet there are no standardized quality measures for programs providing these services (Glied et al. 2015; Substance Abuse and Mental Health Services Administration 2009). In an era increasingly focused on outcomes, healthcare organizations require standardized frameworks by which to measure the quality of the services they provide. Standardized measures are needed for comparisons and benchmarking between programs and to assist organizations in defining goals for internal quality improvement activities. This will become increasingly important as pay-for-performance initiatives expand with healthcare reform. In addition, standardized measures and terminology are needed to support research efforts in crisis operations and quality improvement. In response to these needs, we developed the Crisis Reliability Indicators Supporting Emergency Services (CRISES) framework to guide the creation of a standardized measure set for the programs providing emergency psychiatric and crisis care within our organization, which is the largest provider of facility-based emergency psychiatric care for adults and children in Arizona. We will describe the method used to develop the CRISES framework and the resulting measures. The CRISES framework is a method rather than a static measure set; thus some measures are designated provisional as we continue to evolve improved measures or respond to new customer needs. This framework provides a starting point for the development of standardized measures for the crisis field as a whole.

The term “crisis services” encompass a wide variety of programs and services. These include facility-based psychiatric emergency services, 23-h observation, crisis stabilization beds, crisis respite beds, mobile crisis outreach teams, crisis hotlines, warm lines, peer support, and others. In this work, we use the term “crisis program” to refer to facility-based psychiatric emergency services and 23-h observation. Such services may be delivered in a freestanding behavioral health facility or within a medical ED.

Crisis programs share features in common with emergency departments, urgent care clinics, inpatient psychiatric facilities, and outpatient mental health clinics, yet they are distinctly different. Standards and measures designed for these settings have been variably applied to crisis programs, but they are an imperfect fit. For example, in our own organization, two programs providing identical 23-h observation services have different licenses due to differences in their respective facilities’ physical plant specifications. One is licensed as an inpatient psychiatric unit and the other as an outpatient clinic. As a consequence, the two programs are held to different regulatory and quality standards, neither of which is the best fit for the services provided. This illustrates the need for an independent set of crisis measures that supports a common definition of quality crisis services and allows comparison between similar programs.

We endeavored to develop a measure set that remained under the sphere of influence of an individual crisis program while also reflecting the desired contribution of the crisis program to the functioning of the behavioral health system as a whole. Thus our measures focus on the experience of the individual from the time of arrival to discharge and the interface between the crisis program and its community partners. Such measures have a more narrowed scope than those of managed care organizations (MCOs) and state/local behavioral health authorities (BHAs). At the MCO/BHA level, measures are often designed to assess the functioning of the crisis system as a whole and may not be directly transferable to an individual service provider. For example, it is common for a behavioral health system to measure whether patients discharged from a crisis program are seen by their outpatient behavioral health provider within a certain timeframe, such as 7 days. This measure is designed to incentivize the MCO/BHA to influence the behavior of its contracted providers—both the crisis program and outpatient clinic—in order to meet this metric. While this is a worthwhile measure and all parties should collaborate to ensure that it is met, it is not feasible for a crisis program to be solely responsible. Rather, the crisis program and outpatient clinic should select internal process metrics that facilitate the attainment of this shared goal, such as ensuring that appointments are made and communication occurs between the crisis program and clinic. Then the MCO/BHA can focus on systemic issues that hinder attainment the larger goal.

## Methods

### CTQ Tree

**Fig. 1:**
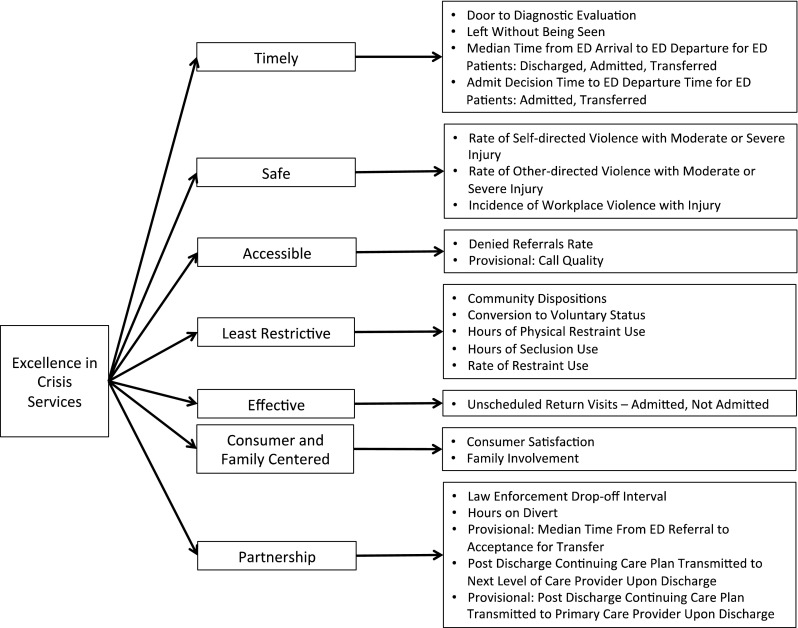
CRISES framework

We began by employing a quality improvement tool called a Critical To Quality (CTQ) Tree. This tool is designed to help an organization translate its values into discrete measures (Lighter and Lighter 2013). When building a CTQ Tree, the first step is to define the value we are trying to accomplish, in this case “Excellence in Crisis Services.” The next step is to define the key attributes that comprise excellent crisis services, from the perspective of the customer. Because a crisis program plays such a vital role in the community, it has many customers and stakeholders. These include the individuals receiving care, law enforcement, emergency departments, other healthcare providers, staff, etc. We defined our key attributes as Timely, Safe, Accessible Least Restrictive, Effective, Consumer/Family Centered, and Partnership (see “Results” section for a detailed description of each). The CRISES domains are consistent with the Institute of Medicine’s six aims for quality healthcare: Safety, Effectiveness, Equity, Timeliness, Patient-centeredness, and Efficiency (Institute of Medicine 2001) while also focusing attention on goals unique to the crisis setting. The CTQ Tree and the resulting CRISES measures are depicted in Fig. 1.

### Measure Selection

Next, we selected discrete measures to reflect the key attributes defined above. Many frameworks exist to inform the selection of quality measures. We employed the criteria described by Hermann and Palmer (2002) which require that measures are meaningful, feasible, and actionable. Some key considerations regarding each of these requirements are outlined below.

#### Meaningful

Does the measure reflect a process that is clinically important? Is there evidence supporting the measure? Compared to other fields, there is a paucity of research on crisis quality measures, so we must rely on face validity or adapt measures for which there is evidence in other settings. The emergency medicine field has put forth much effort in defining standardized measures (Welch et al. 2011), many of which are applicable to crisis services. When possible, measures should be selected or adapted from measures that have been endorsed by organizations that set standards for quality measurement such as the National Quality Forum (NQF), Centers for Medicaid and Medicare Services (CMS), The Joint Commission (TJC), Agency for Healthcare Research and Quality (AHRQ) etc.

#### Feasible

Is it possible to collect the data needed to provide the measure? If so, can this be done accurately, quickly, and easily? Data must be produced within a short timeframe in order to be actionable (see below). An organization’s quality department staff should be able to spend most of its time addressing identified problems rather than performing time-consuming manual chart audits. With the advent of electronic health records (EHRs), it is now possible to design processes that support automated reporting, making it feasible to quickly obtain data that were previously too complex or labor intensive to collect via chart abstraction.

#### Actionable

Do the measures provide direction for future quality improvement activities? Are the factors leading to suboptimal performance within the span of control of the organization to address? A crisis program is in a position to identify problems in the community-wide system of care, and should collaborate with system partners to fix systemic issues. However, its own core measures must be within its sphere of influence to improve, otherwise there is the tendency to blame problems on external factors rather than focus on the problems it can address. Are there established benchmarks towards which to strive? There are few benchmarks for crisis services so we must often rely on internally developed goals, or attempt to benchmark against inpatient psychiatric services or emergency medicine.

## Results

### Descriptive Data

Descriptive data are needed for program and operational design, benchmarking between similar programs, and providing context for performance on quality measures. For example, the emergency medicine field stratifies programs both by volume and by measures of acuity such as Emergency Severity Index (ESI) scores and Intensive Care Unit (ICU) admission rates. Table 1 contains suggested categories for describing the characteristics of crisis programs and the populations they serve.

**Table 1:** Descriptive data

*Population characteristics*
Age
Gender
Race
Ethnicity
Referral source: police, walk-in, child protective custody, etc.
Payer
Legal status: voluntary, involuntary, assisted outpatient treatment, etc.
Housing status
Diagnosis
Co-occurring substance use disorders
Acute substance intoxication or withdrawal
Trauma history
Chronic medical disease (e.g. diabetes, congestive heart failure)
Primary language
*Program characteristics*
Volume: number of encounters annually
Age range served: child, adolescent, adult, geriatric
Law enforcement referral rate: percentage of visits arriving via law enforcement
Involuntary referral rate: percentage of visits arriving under involuntary legal status
Level of care: urgent care, emergency services, 23-h observation, sub-acute crisis stabilization, crisis residential, etc
Locked versus unlocked: Does the program contain a locked unit?
Accessibility: Does the program accept involuntary law-enforcement drop-offs? Does the program require medical clearance at an outside ED or via EMS before arrival?
Hospital setting: Is the program a freestanding behavioral health facility, a program within a medical ED, other?
Community setting: Urban, rural, etc.?
Teaching status: Does the program serve as a training site for residents and medical students?

### CRISES Domains and Measures

The individual measures and their definitions are listed in Table 2. A description of each domain and the rationale for selection of the corresponding measures are below.

**Table 2:** CRISES measures definitions

Measure	Definition	Adapted from existing measure
*Timely*		
Door to Diagnostic Evaluation by a Qualified Behavioral Health Professional	Median time (in minutes) from ED arrival to provider contact	NQF-0498 (CMS OP-20)
Left Without Being Seen	Number of patients who leave the ED without being evaluated by qualified personnel divided by the total number of ED visits	NQF-0499 (CMS OP-22)
Median Time from ED Arrival to ED Departure for Admitted ED Patients	Time (in minutes) from ED arrival to ED departure for patients admitted to the facility from the emergency department	NQF-0496 (CMS ED-1)
Median Time from ED Arrival to ED Departure for Discharged ED Patients	Time (in minutes) from ED arrival to ED departure for patients discharged from the emergency department	NQF-0496 (CMS OP-18)
Median Time from ED Arrival to ED Departure for Transferred ED Patients	Time (in minutes) from ED arrival to ED departure for patients transferred to an outside facility from the emergency department	NQF-0496 (CMS OP-18)
Admit Decision Time to ED Departure Time for Admitted Patients	Median time (in minutes) from admit decision time to time of departure from the emergency department for patients admitted to the facility from the emergency department.	NQF-0495 (CMS ED-2)
Admit Decision Time to ED Departure Time for Transferred Patients	Median time (in minutes) from admit decision time to time of departure from the emergency department for patients transferred to an outside facility from the emergency department	NQF-0495 (CMS ED-2)
*Accessible*		
Denied Referrals Rate	Percent of referrals denied admission to the crisis program for any reason other than overcapacity	No
Provisional: Call Quality	Composite score on “mystery caller” assessment tool	No
*Safe*		
Rate of Self-directed Violence with Moderate or Severe Injury	Number of incidents of SDV with moderate or severe injury per 1000 visits	Uses CDC methodology
Rate Other-directed Violence with Moderate or Severe Injury	Number of incidents of violence to other persons receiving care with moderate or severe injury per 1000 visits	Uses CDC methodology
Incidence of Workplace Violence with Injury	Total number of incidents of workplace violence to staff resulting in injury divided by the total number of hours worked	Uses OSHA methodology
*Least*-*Restrictive*		
Community Dispositions	Percentage of visits resulting in discharge to community-based setting	No
Conversion to Voluntary Status	Percentage of involuntary arrivals requiring admission/transfer to inpatient care that are admitted/transferred under voluntary status	No
Hours of Physical Restraint Use	The total number of hours that all patients were maintained in physical restraint per 1000 patient hours	NQF-0640 (HBIPS-2)
Hours of Seclusion Use	The total number of hours that all patients were maintained in seclusion per 1000 patient hours	NQF-0641 (HBIPS-3)
Rate of Restraint Use	Total number of restraint episodes per 1000 visits	No
*Effective*		
Unscheduled Return Visits—Total	Percentage of discharges that resulted in an unscheduled return visit	No
Unscheduled Return Visits—Not Admitted	Percentage of discharges that resulted in an unscheduled return visit in which the return visit did not result in admission or transfer to an inpatient psychiatric facility	No
Unscheduled Return Visits—Admitted	Percentage of discharges that resulted in an unscheduled return visit in which the return visit resulted in admission or transfer to an inpatient psychiatric facility	No
*Consumer and Family Centered*		
Consumer Satisfaction	Likelihood to recommend	IHI Experience of Care
Family Involvement	Percentage of individuals for whom there is either a documented attempt to contact family/other supports or documentation that the individual was asked and declined consent to contact family/other supports	No
*Partnership*		
Law Enforcement Drop-off Interval	Time (in minutes) from law enforcement arrival to law enforcement departure	EMS Offload Interval
Hours on Divert	Percentage of hours the crisis center was unable to accept transfers from medical EDs due to overcapacity	No
Provisional: Median Time from ED Referral to Acceptance for Transfer to the Crisis Program	Time (in minutes) from initial contact from the referring ED to notification that the patient has been accepted for transfer to the crisis program	No
Post Discharge Continuing Care Plan Transmitted to Next Level of Care Provider Upon Discharge	Percentage of discharges in which the continuing care plan was transmitted to the next level of care provider	NQF-0558 (HBIPS-7)
Provisional: Post Discharge Continuing Care Plan Transmitted to the Primary Care Provider Upon Discharge	Percentage of discharges in which the continuing care plan was transmitted to the primary care provider	NQF-0558 (HBIPS-7)

#### Timely

Timeliness is especially critical in the crisis setting. CMS has developed measures to assess throughput in emergency departments (Centers for Medicare and Medicaid Services 2015a, c) and performance on these measures is now publicly available on its Hospital Compare website http://hospitalcompare.hhs.gov. The CMS ED throughput measures are directly applicable to the crisis setting and we have adopted them with only minor modification.

#### Accessible

A crisis program must be accessible to the community at all times and welcome anyone in need of services. However, many crisis programs are not subject to the Emergency Medical Treatment and Active Labor Act (EMTALA), and some have created barriers to access such as overly rigorous exclusion criteria. Thus we include a measure of the percentage of referrals that are denied admission for any reason other than overcapacity. In addition, we are developing a “mystery caller” assessment tool (O’Neill et al. 2012) to assess our customer service and determine whether callers to the crisis program get their needs met in a welcoming, respectful, and timely manner.

#### Safe

A core function of crisis services is to address potential dangerousness to self or others. Regulatory reporting requirements for incidents of self-harm within the facility often include vague qualifiers such as “serious suicide attempt” that leave much to interpretation. The Centers for Disease Control (CDC) has proposed a classification system for self-directed violence (SDV) that allows for more precise descriptions of the behaviors and resulting injuries (Crosby and Melanson 2011). Using this system, we measure incidents of SDV (suicidal or non-suicidal) with moderate or severe injury. For episodes of violence towards other persons receiving care, we include other-directed violence with injury using the classification system for SDV described above. In addition to the need for patient safety, there has been increasing awareness of the high prevalence of workplace violence towards healthcare workers, especially in EDs and behavioral health facilities (Anderson and West 2011; Gacki-Smith et al. 2009). For violence towards staff, we include a measure based on the methodology outlined by the Occupational Safety and Health Administration (OSHA) for measuring the incidence of workplace violence with injury (Occupational Safety and Health Administration).

#### Least Restrictive

A crisis program should strive to resolve the crisis in partnership with individuals and their supports such that the majority can continue their recovery in the least restrictive setting possible. Thus we measure the percentage of visits that result in discharge to a community setting and the percentage of involuntary arrivals requiring inpatient admission that are converted to voluntary status. Measures of restraint use are an important indicator of the use of less restrictive interventions within the facility. The Joint Commission Hospital Based Inpatient Psychiatric Services (HBIPS) measures (Joint Commission on Accreditation of Healthcare Organizations 2012a) include two items (HBIPS-2 and HBIPS-3) that reflect the duration of physical restraint and seclusion use expressed as hours of each per 1000 patient hours. State and national benchmarks for inpatient units are available at http://qualitycheck.org and CMS has incorporated the HBIPS measures into its Inpatient Psychiatric Facility Quality Reporting (IPFQR) Program (Centers for Medicare and Medicaid Services 2015c). In contrast, there is no standard methodology for reporting the rate of restraint occurrences. We have defined an “event” as the single application of a restraint (e.g. physical hold, mechanical restraint, or seclusion) and an “episode” as the continuous restriction of a person’s freedom of movement via the use of one or more restraint events and express the rate as episodes per 1000 visits.

#### Effective

Crisis services may be considered effective when the individual had his/her needs met and leaves with a plan that facilitates the continuation of recovery in the community setting. The most readily available proxy metric would then be unscheduled return visits (URV), based upon the assumption that the need to return to the crisis program represents a failure of the discharge plan. We measure URV within 72 h, as this timeframe is becoming more common in the ED literature (Trivedy and Cooke 2015) and is consistent with the Joint Commission’s timeframe in which a hospital is held accountable for suicide post-discharge. There is emerging evidence suggesting that all URVs are not equal (Hu et al. 2012). One group is comprised of individuals who are discharged from an ED, return to the ED, and are then discharged again. For this group, the URV may represent opportunities for improvement within the crisis program but may also indicate problems with community services that it is unable to address without help from system partners. In contrast, individuals who are discharged from an ED, return to the ED, and are then admitted to an inpatient unit on their second visit may—but not necessarily—represent an error in decision-making. Thus we measure these two types of URV separately.

#### Consumer and Family Centered

We have adapted surveys from psychiatric inpatient and medical ED settings to measure consumer satisfaction at our programs and use the anchor question “likelihood to recommend” to serve as a proxy for overall satisfaction with the healthcare service received (Stiefel and Nolan 2012). In addition, families often play a critical role in crisis resolution (Substance Abuse and Mental Health Services Administration 2009) and thus we assess whether there is documentation that our staff attempted to involve family or other supports in the care of the individual in crisis.

#### Partnership

##### Partnerships with Law Enforcement

Individuals with mental illness are disproportionately represented in the criminal justice system (James 2006), and we have worked very closely with law enforcement to divert individuals with behavioral health needs into more appropriate treatment settings. We have learned that in order to achieve this goal we must be as user friendly as possible to law enforcement; thus, we measure law enforcement drop-off time and strive for a target of 10 min. This measure is analogous to the ED process metric of EMS offload interval—arrival time to the time the patient is removed from the ambulance stretcher and care is assumed by the ED staff. Similarly, our goal is to transfer the individual from police custody to the care of the crisis center staff as quickly as possible.

##### Partnerships with EDs

Boarding of psychiatric patients in medical EDs is an increasing problem for the healthcare system. Crisis programs are poised to help EDs mitigate the burden of psychiatric boarding (Little-Upah et al. 2013; Zeller et al. 2014) and should develop measures reflecting this value. The Joint Commission has recently required EDs to measure the time from decision-to-admit to the actual admission time (Joint Commission on Accreditation of Healthcare Organizations 2012c). Perhaps in the future it will be possible to use that data to construct a composite measure of a community’s total psychiatric boarding. While such a measure could inform system planning, more feasible and actionable measures for a crisis program are those that reflect its accessibility to EDs. We currently measure the percentage of time the crisis program is unable to accept transfers from outside EDs due to overcapacity (i.e. diversion). We are also developing a measure assessing the time from ED request for transfer to the crisis program’s communication that the patient has been accepted for transfer.

##### Partnerships with Other Care Providers

We have adopted the HBIPS-7 measure regarding the transmittal of a post-discharge continuing care plan to the next level of care provider and are developing a similar measure reflecting transmittal of key information to the primary care provider.

## Discussion

We developed the CRISES framework in response to our own organizational needs and have used it to guide the creation of quality measures that inform internal performance improvement initiatives and facilitate comparison of performance across programs. The framework is comprised of two components—the CRISES domains and the measures supporting each domain. The CRISES domains are consistent with the IOM’s six aims for quality healthcare while also focusing attention on goals unique to the crisis setting, such as least-restrictive care and community partnerships. We attempted to limit the number of measures to a manageable number and thus some potentially useful measures were excluded. In particular, we did not include measures that track whether or not a particular type of screening or assessment was performed. Rather, we prefer to evaluate the content of clinical assessments and perform qualitative reviews on a random sampling of charts and then provide individual feedback via our clinical supervision and peer review processes. Other limitations of this work are that these measures have not been endorsed for use in the crisis setting by professional or healthcare quality improvement organizations and they have only been tested within our own crisis programs.

### Implementation and Application

The CRISES measures form the foundation of the quality scorecards in use at our facilities. It took approximately 1 year to build our first scorecard due to challenges with EHR reporting capabilities that required repeated cycles of data validation via manual chart audits, changes to our documentation processes, and staff education. Having learned from this experience, we specified reporting capability for these measures as a contract deliverable with our EHR vendor as they transition another of our facilities to electronic charting.

We have hardwired ongoing assessment of the validity and utility of these measures into our routine quality and operational processes. For example, the scorecard is reviewed at monthly quality meetings. Specific measures such as URV are tracked and trended in monthly utilization management meetings; when indicated, individual cases are reviewed and referred for internal peer review or to the relevant outpatient clinic or system partner. Law enforcement drop-off time data is reviewed at monthly meetings with local law enforcement. Individual employee injuries and incidents of self/other directed violence are reviewed in daily operational huddles and tracked and trended in monthly restraint committee meetings.

**Fig. 2:**
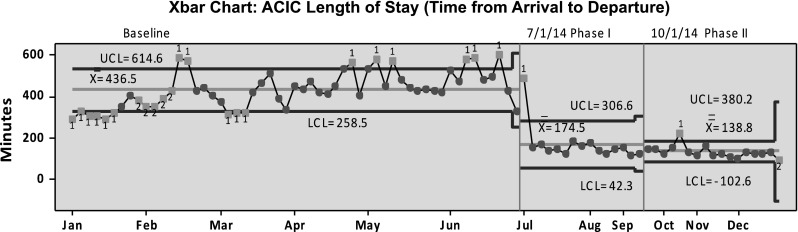
Improvement in time from arrival to departure. Change in time from arrival to departure in response to two phases of process improvements. ACIC, Adult Crisis Intervention Clinic; Xbar, sample mean; UCL, upper control limit; LCL, lower control limit

We have successfully used CRISES measures as outcomes for process improvement initiatives within our organization. As an example, Fig. 2 depicts a control chart showing improvements in the Time from Arrival to Departure in one of our crisis urgent care clinics in response to two phases of process improvements. In addition, at that facility we have achieved a 78 % decrease in Door to Diagnostic Evaluation and a 60 % decrease in staff injuries (Balfour et al. 2014). The CRISIS measures have also proven useful in discussions with our payers regarding new state requirements for Pay for Performance contracting. Our work in this area has allowed us to proactively propose sensible metrics for which we already have established baseline performance.

### Future Directions

We anticipate that the individual CRISES measures will continuously evolve. Our work has highlighted the need for further research and consensus on certain definitions and assessment tools. As the crisis field advances and new customer needs are identified, new and improved measures will be developed and measures that are no longer useful will be retired. However, the CRISES domains will continue to be a guidepost to inform the development of additional measures. For example, after the creation of the CRISES framework, we recognized that the Partnership domain would be enhanced by the inclusion of a measure reflecting partnership with primary care providers, and now a new provisional measure is in development. Although we started with measures based on existing standards, we continue to develop improved standards. For example, in order to drive more proactive care coordination, we are exploring a measure requiring notification to the outpatient mental health provider within 1 h of arrival. Such a measure may eventually accompany or supplant the current HBIPS-7 measure. Similarly, we are exploring measures to drive more proactive efforts to identify those who need connection to a primary care provider.

The measures included here focus on the internal operations supporting the care of an individual receiving service at a facility-based psychiatric emergency program. While some of the CRISES measures may be generalizable across all crisis settings, different measures may be required for other levels of care and types of programs. Regardless of setting, future measure development should include emphasis on how crisis programs support the community and fit within the larger system of care. Future measures may assess how well crisis programs accept continuing responsibility once the individual leaves its walls (e.g. measures assessing collaboration with outpatient providers for high utilizers, outreach during the gap between discharge and follow-up care, scheduled return visits for individuals unable to obtain timely follow-up appointments, etc.). Organizational assessments could provide more detailed measures of accessibility and capability such as exclusion criteria, pre-admission medical clearance requirements, detoxification protocols, staff competencies, etc.

Healthcare providers will be increasingly required to demonstrate their value as we continue to strive towards achieving the Triple Aim of improving patient experience, population health, and cost (Berwick et al. 2008; Glied et al. 2015). The CRISES framework provides a way for behavioral health crisis programs to select measures that demonstrate value to multiple customers using language and methods familiar to industry and quality leaders. Quality measures and pay for performance targets are not yet well defined for behavioral health, and even less so for crisis services. We in the crisis field have an exciting but time-limited opportunity to define our own standards for the unique services we provide.
